# Supplementary material for: Effect of Drying Methods on Lutein Content and Recovery by Supercritical Extraction from the Microalga Muriellopsis sp. (MCH35) Cultivated in the Arid North of Chile
Source: Mar Drugs. 2020 Oct 26;18(11):528. doi: 10.3390/md18110528 (PMC7692189; doi:10.3390/md18110528)
Supplement: Supplementary file 1 [file marinedrugs-18-00528-s001.pdf]

Supplementary material -Table S1. The following is available as Table S1: Regression coefficients (values of variables are specified in their original units) extraction yields (Y), lutein content and recoveries using spray- (SD) and freeze-dried (FD) biomass, statistics for the fit obtained by multiple linear regression.

	**Spray-dried biomass**						**Freeze-dried biomass**					
**Terms of the model**	**Y**		**Lutein content**		**Lutein recovery**		**Y**		**Lutein content**		**Lutein recovery**	
	**Estimated**	***P-value***	**Estimated**	***P-value***	**Estimated**	***P-value***	**Estimated**	***P-value***	**Estimated**	***P-value***	**Estimated**	***P-value***
constant	0.881		39.010		32.056		7.835		39.017		8.056	
A: T	0.304	0.681	-0.353	0.964	0.827	0.441	0.185	0.881	2.520	0.583	4.578	0.435
B: P	-0.055	0.192	4.135	0.599	-0.294	0.343	-0.049	0.236	14.778	0.018*	-0.612	0.823
C: Ethanol	0.490	0.020*	22.623	0.028*	2.476	0.001*	0.096	0.021*	35.863	$4.0E^{-4}$*	0.429	0.006*
AA	-0.003	0.396	-0.880	0.939	-0.008	0.461	-0.003	0.470	6.426	0.356	-0.033	0.365
AB	$3.67E^{-5}$	0.936	0.975	0.929	$3.72E^{-4}$	0.810	$1.57E^{-4}$	0.749	-13.520	0.077	-0.002	0.647
AC	$1.44E^{-4}$	0.962	-3.840	0.728	$1.67E^{-4}$	0.987	0.002	0.504	3.910	0.548	0.019	0.584
BB	$7.73E^{-5}$	0.307	2.055	0.857	$3.51E^{-4}$	0.185	$4.48E^{-5}$	0.563	8.711	0.227	0.001	0.299
BC	-0.001	0.062	11.965	0.303	-0.002	0.336	$-1.22E^{-4}$	0.803	5.155	0.435	0.002	0.644
CC	0.001	0.823	-47.010	0.008*	-0.040	0.011*	-0.002	0.559	-32.699	0.004*	-0.028	0.430
Lack-of-fit		0.002		0.329		0.487		0.057		0.066		0.049
					Statistics for the goodness of fit of the model							
R^2	0.815		0.858		0.928		0.751		0.960		0.837	
Adjusted R^2	0.481		0.603		0.799		0.304		0.888		0.544	
RSD	1.305		10.424		4.389		1.388		6.076		14.354	
P	0.403		0.783		0.338		0.033		0.232		0.018	

Note: R^2–determination coefficient, adjusted R^2, RSD–residual standard deviation, *P-value* of the lack-of-fit test for the model; *- significant coefficients of the model, T–Temperature, P–Pressure, Ethanol–Co-solvent
